# Supplementary material for: Analysis of subcellular transcriptomes by RNA proximity labeling with Halo-seq
Source: Nucleic Acids Res. 2021 Dec 7;50(4):e24. doi: 10.1093/nar/gkab1185 (PMC8887463; doi:10.1093/nar/gkab1185)
Supplement: gkab1185_Supplemental_Files [file gkab1185_supplemental_files.zip › SUPPLEMENTARY DATA LEGENDS.docx]

**SUPPLEMENTARY DATA LEGENDS**

**Table S1.** RNAseq enrichments (pulldown / input) for the Halo-p65 Haloseq experiment, as identified by DESeq2. Only genes with at least 5 counts in all p65 samples are included. Gene biotype designations are drawn from biomaRt.

**Table S2.** RNAseq enrichments (pulldown / input) for the H2B-Halo Haloseq experiment, as identified by DESeq2. Only genes with at least 5 counts in all H2B samples are included. Gene biotype designations are drawn from biomaRt.

**Table S3.** RNAseq enrichments (pulldown / input) for the Fibrillarin-Halo Haloseq experiment, as identified by DESeq2. Only genes with at least 5 counts in all fibrillarin samples are included. Gene biotype designations are drawn from biomaRt.

**Table S4.** Changes in H2B-Halo pulldown enrichment following LMB treatment. Log2FC columns represent the genes enrichment in the H2B-Halo pulldown sample compared to input samples. Enrichment change represents the change in this enrichment between LMB treated and untreated samples (treated - untreated). The p value column has been adjusted for multiple hypothesis testing and is the result of tests asking whether the treated and untreated log2FC values were different. This table was modified from the output of Xtail software[(58)](https://paperpile.com/c/dyXB5C/DB8ce).

**Figure S1.** (A, B) Alkynylated molecules can be visualized *in situ* by fusing them with fluorophores (e.g. Cy5-azide) using Click chemistry. Alkynylated molecules are restricted to the cytoplasm in cells containing Halo-p65 (A) and the nucleolus in cells containing Halo-Fibrillarin (B). They are only detectable in cells treated with both DBF and Cy5-azide, demonstrating the ability of HaloTag-restricted DBF to induce alkynylation of biomolecules. (C) Principal component analysis of gene expression values from the Halo-seq histone H2B experiment. (D) Principal component analysis of gene expression values from the Halo-seq p65 experiment. (E) Read coverage over *CXCL2* in Halo-seq histone H2B and p65 experiments. Note the higher intronic coverage in the H2B pulldown sample relative to the H2B input sample, whereas the p65 pulldown sample has less intronic coverage relative to the p65 input sample. (F) As in E, but for the gene *HOXA11*.

**Figure S2**. (A) Principal component analysis of gene expression values from the Halo-seq fibrillarin experiment. (B) Enrichments of RNAs derived from the mitochondrial chromosome in the H2B, p65, and Fibrillarin Halo-seq experiments. All significance tests were performed using a Wilcoxon rank-sum test. p value notation: * < 0.05, ** < 0.01, *** < 0.001, **** < 0.0001.

**Figure S3**. (A) Expression of miniSOG-2 Halo fusion in HeLa cells. Halo fusion proteins were visualized through the addition of a fluorescent Halo ligand to lysate prior to electrophoresis. (B) Expression of APEX2-Halo fusion in HeLa cells. Halo fusion proteins were visualized through the addition of fluorescent Halo ligand to lysate prior to electrophoresis. (C) Spearman correlation of gene-wise enrichment values (streptavidin pulldown / input) for Halo-seq and APEX-seq samples. (D) Spearman correlation of gene-wise enrichment values (streptavidin pulldown / input for Halo-seq; biochemical fraction / total for CeFra-seq) for Halo-seq and CeFra-seq samples.

**Figure S4**. (A) RNA biotinylation, as assayed by RNA dot blot on Halo-seq histone H2B samples with and without LMB treatment. (B) Selected genes whose RNAs were specifically enriched or depleted in the Halo-seq histone H2B experiment following LMB treatment. (C) RNA sequence motifs enriched in the 3′ UTRs of genes that become more enriched in the nucleus following LMB treatment. (D) Changes in H2B pulldown enrichment upon LMB treatment for genes that either do or do not contain AU-rich elements in their 3′ UTRs. (E) RBP binding motif enrichment in the 3′ UTRs of genes that become more enriched in the nucleus following LMB treatment. RBP binding motifs were defined using data from RNA bind-n-seq[(68)](https://paperpile.com/c/dyXB5C/vr9yN), and their enrichments were calculated using FeatureReachR.
