# Supplementary figures and images for: Analysis of subcellular transcriptomes by RNA proximity labeling with Halo-seq
Source: Nucleic Acids Res. 2021 Dec 7;50(4):e24. doi: 10.1093/nar/gkab1185 (PMC8887463; doi:10.1093/nar/gkab1185)

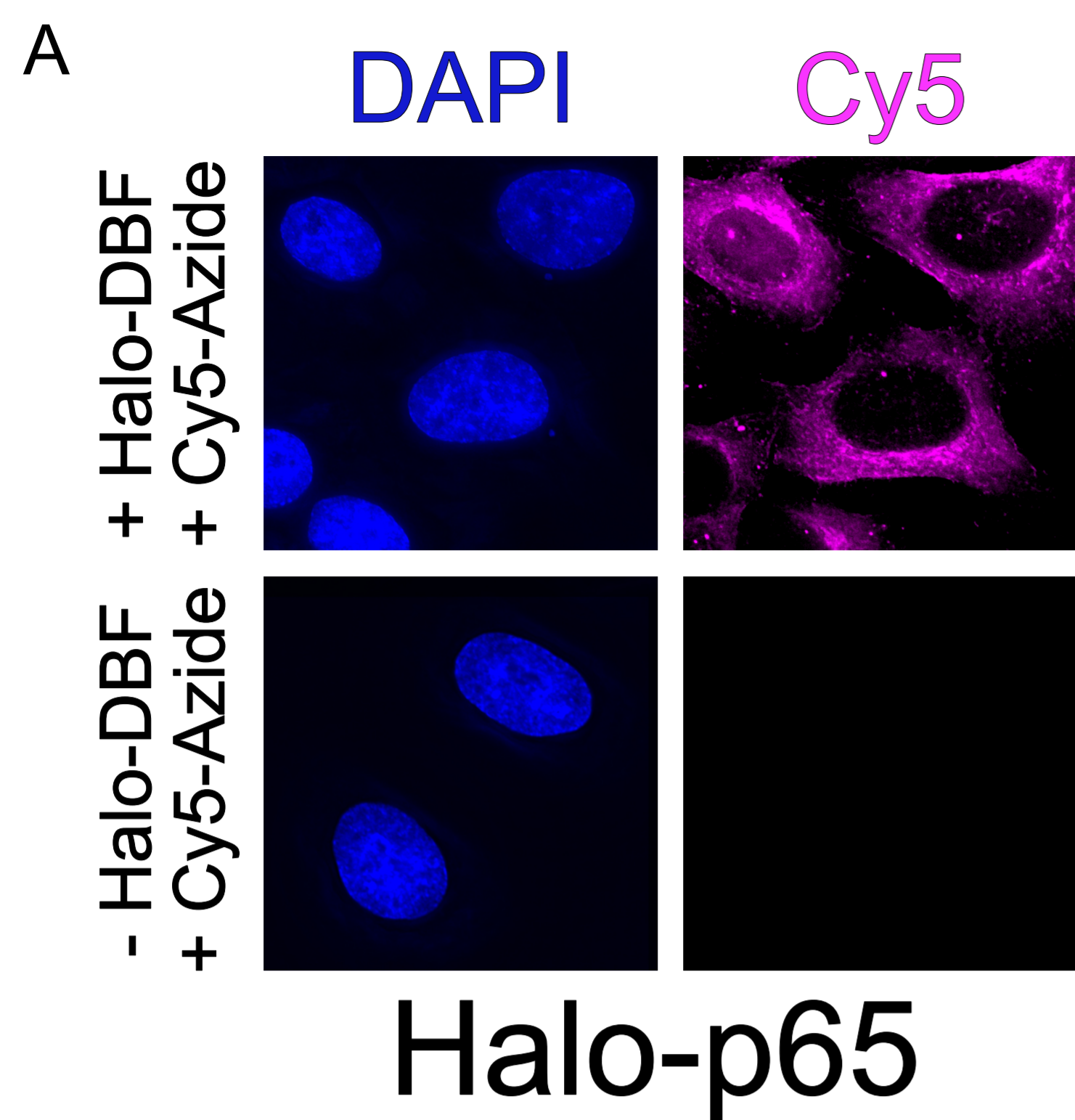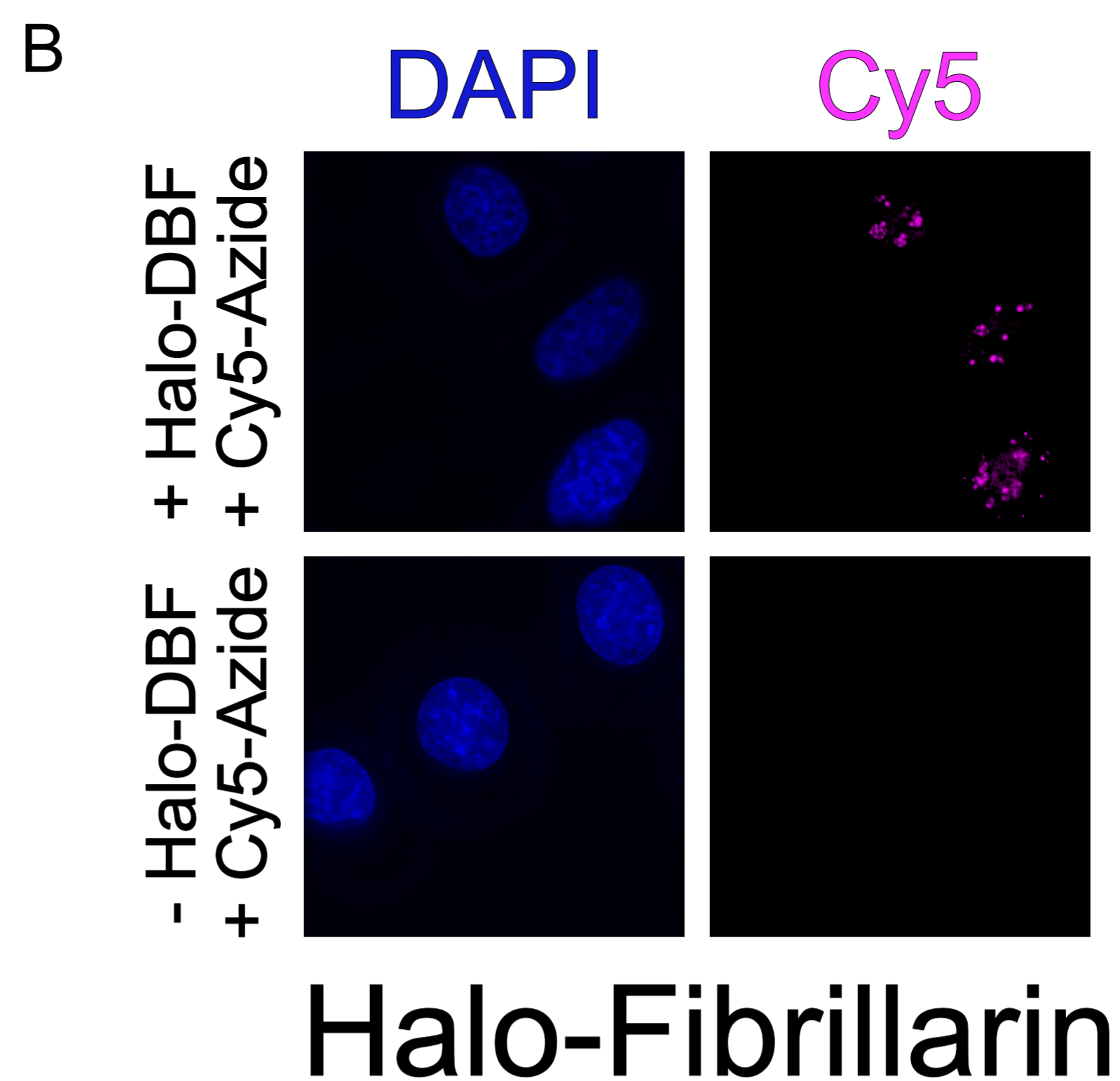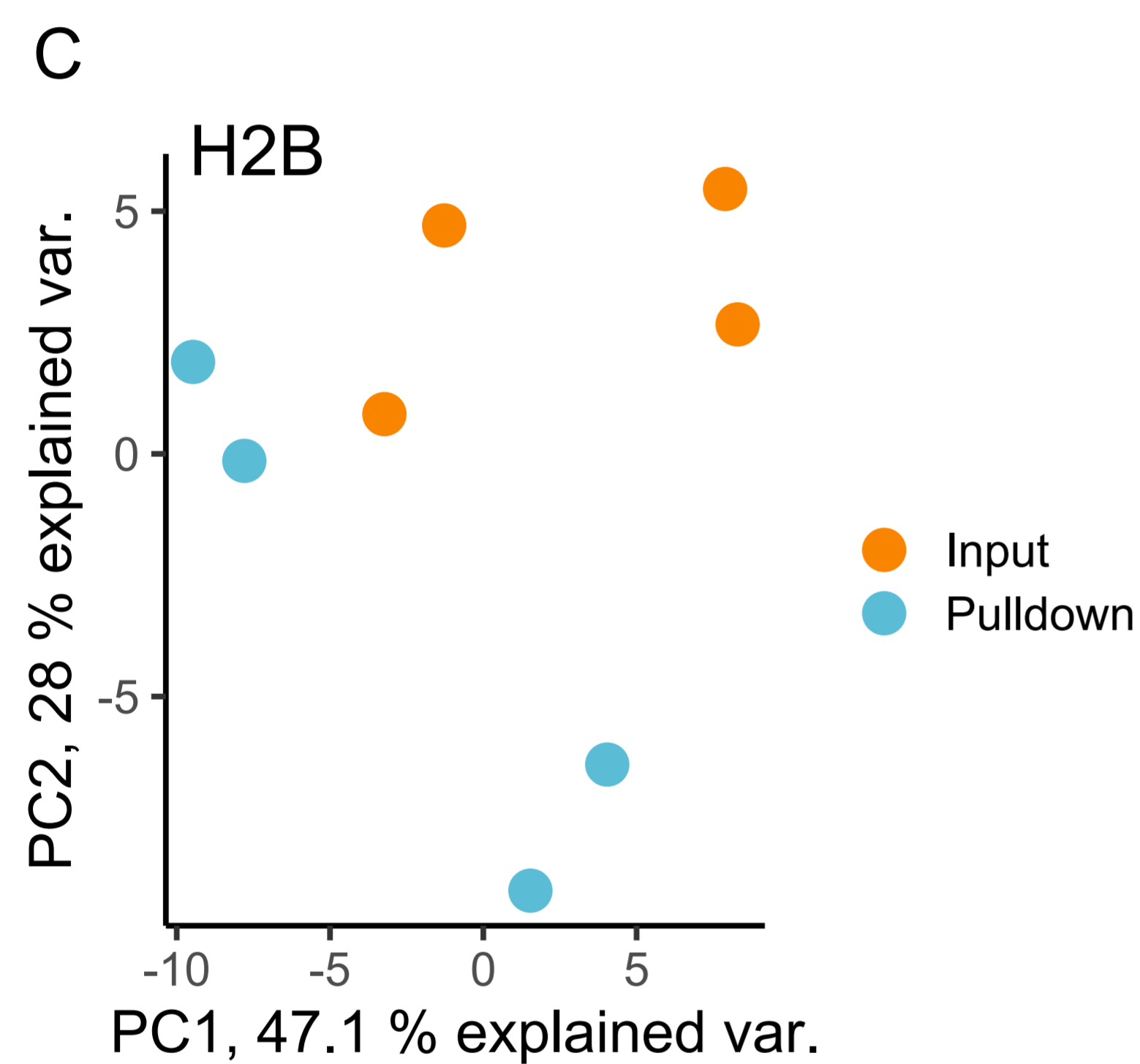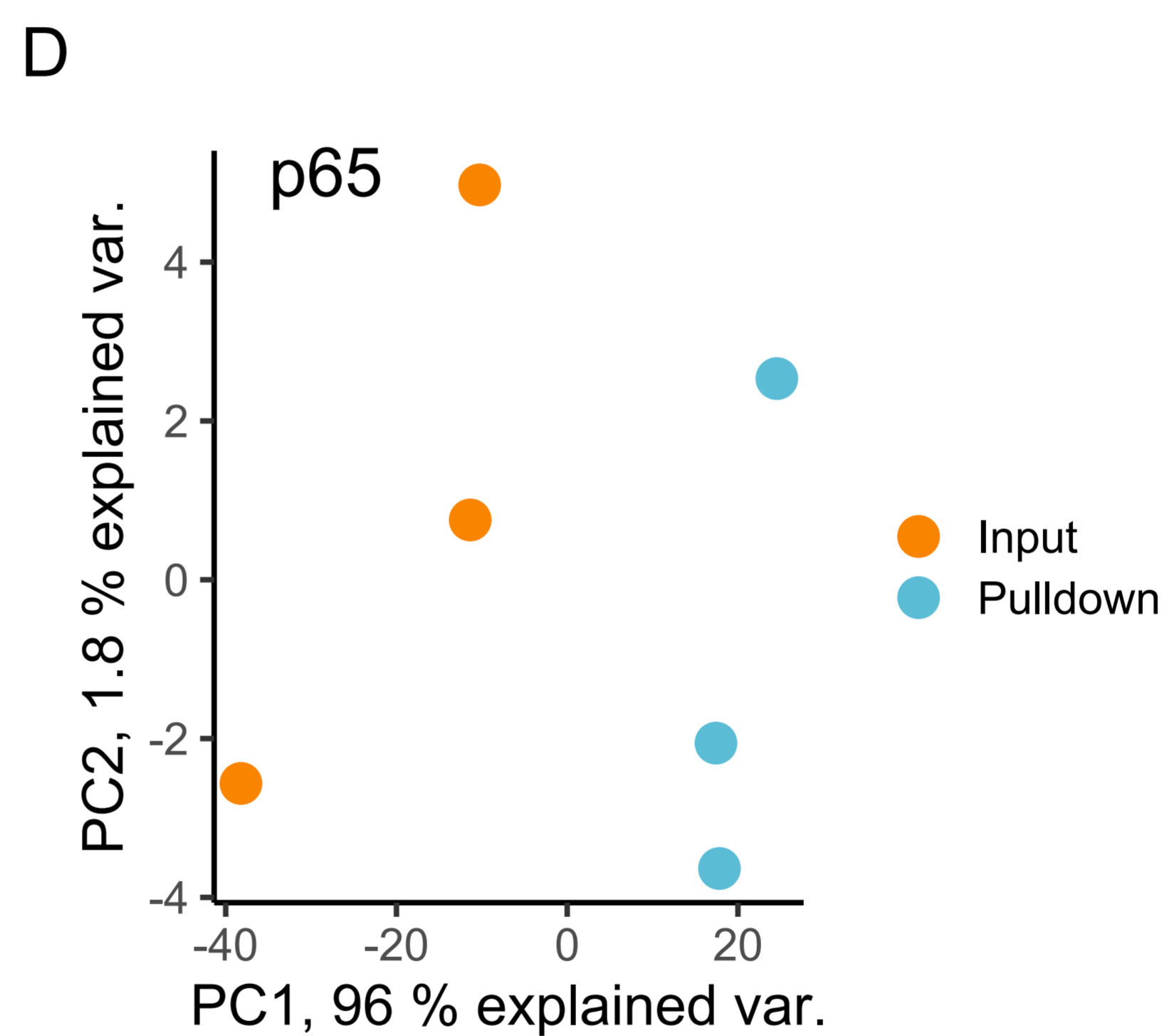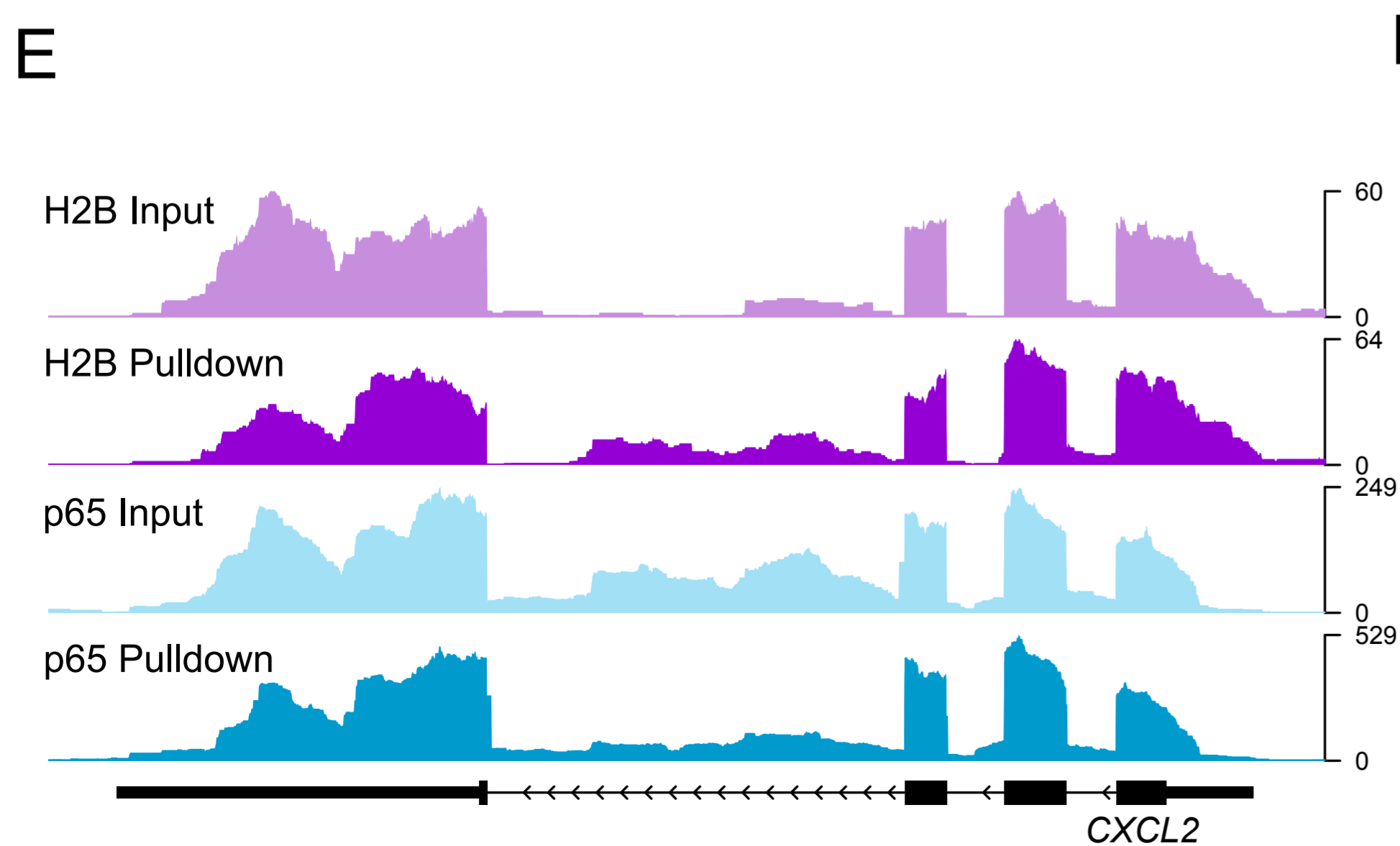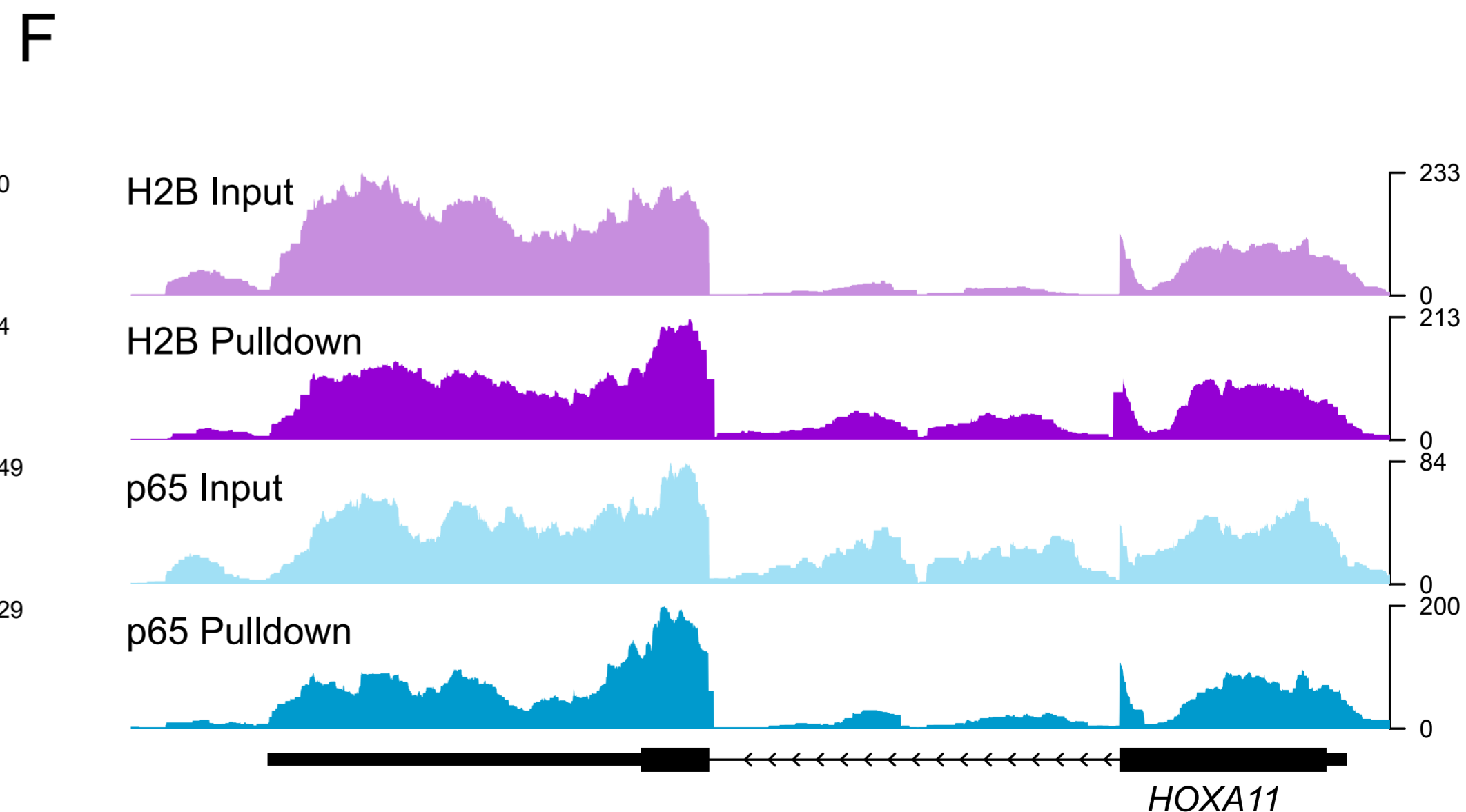

Figure S1

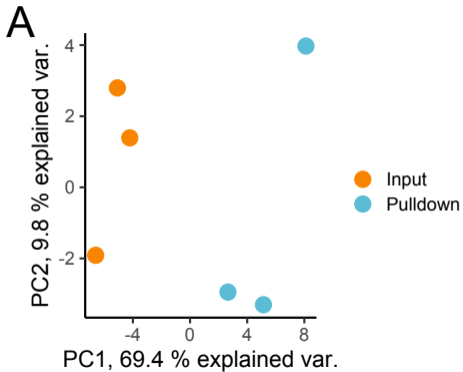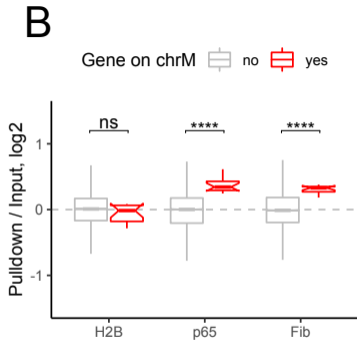

Figure S2

A

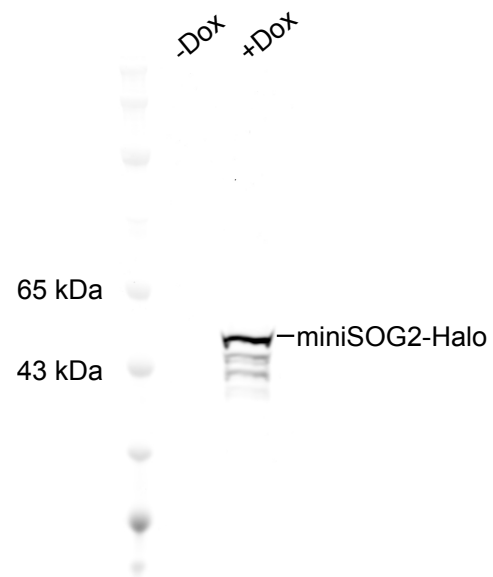

B

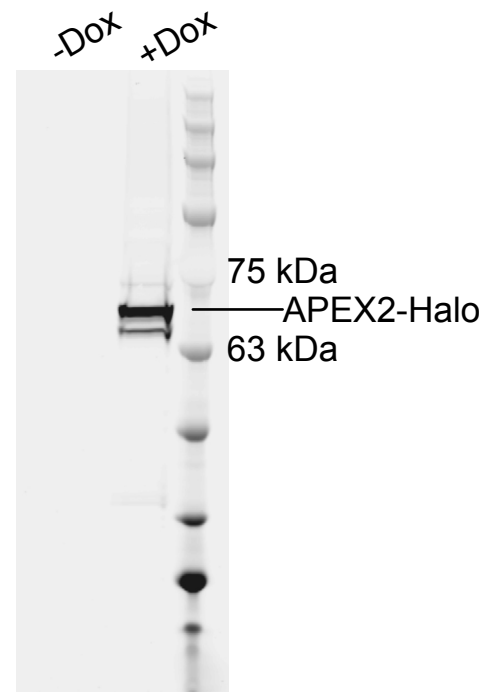

C

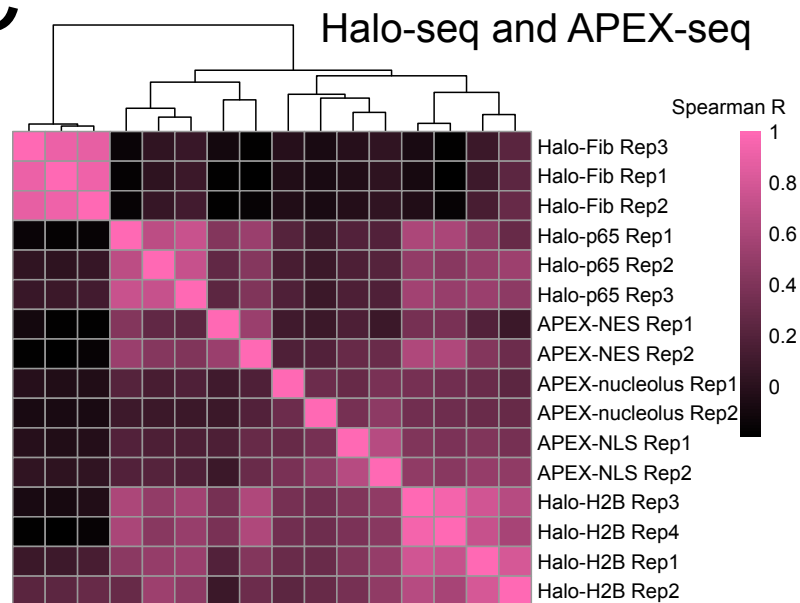

D

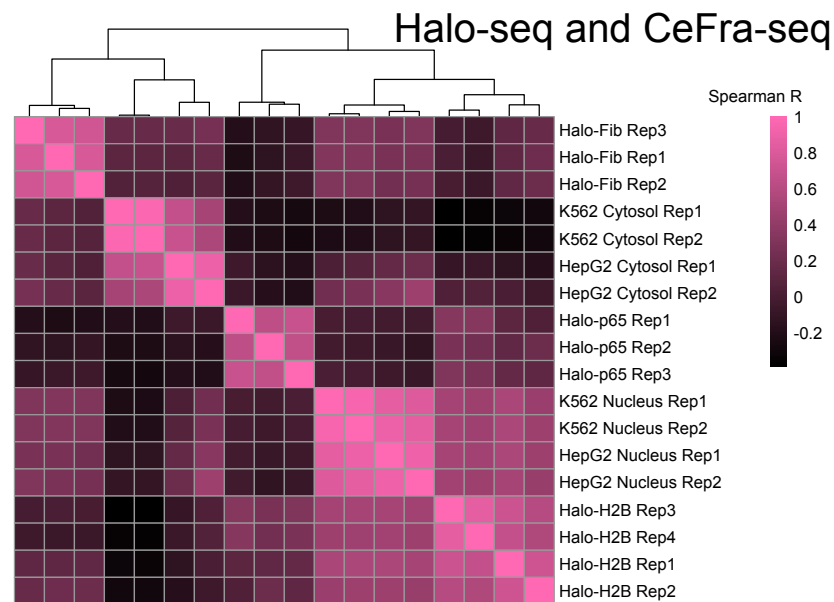

Figure S3

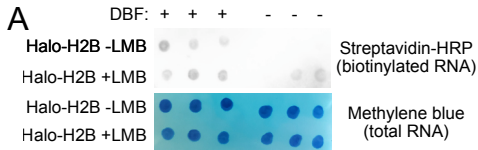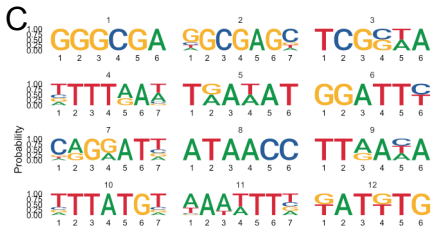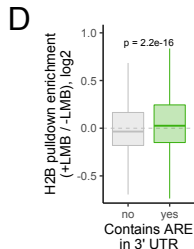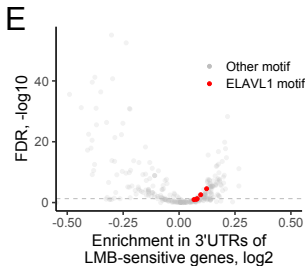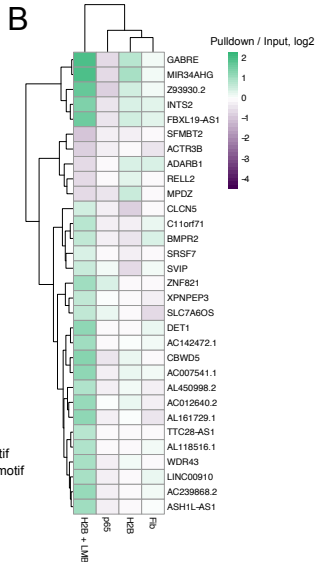

Figure S4

Supplement: gkab1185_Supplemental_Files [file gkab1185_supplemental_files.zip › SupplementaryFigures.pdf]
